# Complete nucleotide sequence and comparative genomic analysis of microcin B17 plasmid pMccB17

**DOI:** 10.1002/mbo3.1402

**Published:** 2024-03-05

**Authors:** Mayokun Ajeigbe, Stephen Childs, Timothy A. Paget, Lewis E. H. Bingle

**Affiliations:** ^1^ School of Nursing and Health Sciences, Faculty of Health Sciences and Wellbeing University of Sunderland Sunderland UK; ^2^ School of Pharmacy & Pharmaceutical Sciences, Faculty of Health Sciences and Wellbeing University of Sunderland Sunderland UK; ^3^ School of Medicine, Faculty of Health Sciences and Wellbeing University of Sunderland Sunderland UK

**Keywords:** Enterobacteriaceae, genome, microcin, plasmid

## Abstract

We present a comprehensive sequence and bioinformatic analysis of the prototypical microcin plasmid, pMccb17, which includes a definitive sequence for the microcin operon, *mcb*. Microcin B17 (MccB17) is a ribosomally synthesized and posttranslationally modified peptide produced by *Escherichia coli*. It inhibits bacterial DNA gyrase similarly to quinolone antibiotics. The *mcb* operon, which consists of seven genes encoding biosynthetic and immunity/export functions, was originally located on the low copy number IncFII plasmid pMccB17 in the *Escherichia coli* strain LP17. It was later transferred to *E. coli* K‐12 through conjugation. In this study, the plasmid was extracted from the *E. coli* K‐12 strain RYC1000 [pMccB17] and sequenced twice using an Illumina short‐read method. The first sequencing was conducted with the host bacterial chromosome, and the plasmid DNA was then purified and sequenced separately. After assembly into a single contig, polymerase chain reaction primers were designed to close the single remaining gap via Sanger sequencing. The resulting complete circular DNA sequence is 69,190 bp long and includes 81 predicted genes. These genes were initially identified by Prokka and subsequently manually reannotated using BLAST. The plasmid was assigned to the F2:A‐:B‐ replicon type with a MOBF12 group conjugation system. A comparison with other IncFII plasmids revealed a large proportion of shared genes, particularly in the conjugative plasmid backbone. However, unlike many contemporary IncFII plasmids, pMccB17 lacks transposable elements and antibiotic resistance genes. In addition to the *mcb* operon, this plasmid carries 25 genes of unknown function.

## Graphical abstract

We have determined the complete sequence of plasmid pMccB17 (isolated in 1977), which carries the prototypical microcin operon *mcb*. The plasmid is 69,190 bp and assigned to the IncFII group of F2:A‐:B‐ replicon type with a conjugation system from the MOBF12 group. While the *mcb* operon is the major noteworthy feature, pMccB17 is unlike many contemporary IncFII plasmids in that it lacks transposable elements and antibiotic resistance genes.

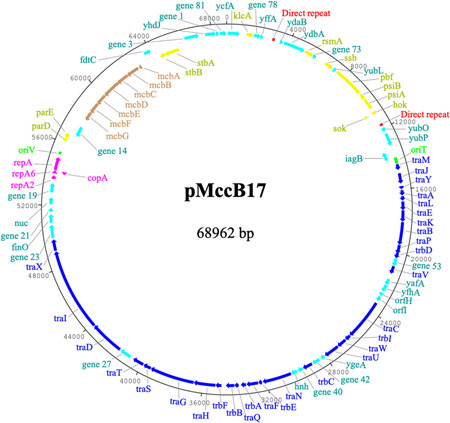

## 1 INTRODUCTION

Microcin B17 (MccB17), is a small (3093 Da), ribosomally synthesized and posttranslationally modified peptide (RiPP) that is produced by *Escherichia coli* (Arnison et al., 2013; Duquesne et al., 2007). This antimicrobial natural product inhibits the class II topoisomerase DNA gyrase in a similar way to quinolone antibiotics while differing slightly in that MccB17 targets gyrase subunit B, whereas quinolones bind gyrase subunit A (Heddle et al., 2001; Pierrat & Maxwell, 2003). Biosynthesis of MccB17 is carried out by the products of 7 genes *(mcbA–G)* that encode precursor peptide, synthase, immunity, and export functions and are arranged in an operon found on the plasmid pMccB17 (Garrido et al., 1988; Genilloud et al., 1989; Yorgey et al., 1994). The 69 amino acid promicrocin (precursor peptide) McbA is posttranslationally modified by the synthetase complex McbBCD, resulting in the formation of heterocycles (thiazole and oxazole) in the sequence. This modified peptide sequence is exported from the host cytoplasm by an efflux pump encoded by *mcbE* and *mcbF*. The last gene, *mcbG* (the product of which binds to the host DNA gyrase), together with *mcbE* and *mcbF* functions to offer immunity to the host cell (Collin et al., 2013). MccB17 is one of the best‐studied microcins and is gaining recognition as a promising template for developing new antibacterial agents (Collin & Maxwell, 2019; Ghilarov et al., 2019; Withanage et al., 2013)

Conjugative plasmid pMccB17, previously known as pRYC17, was originally found in *E. coli* strain LP17 isolated from the intestinal tract of a healthy newborn at Hospital La Paz, Spain, and transferred by conjugation to *E. coli* K‐12 (Baquero et al., 1978). This is a low copy number plasmid (approximately two copies per chromosome) belonging to the IncFII group that includes the archetypes R100 and R1. Plasmid pMccB17 is not known to possess any conventional antibiotic resistance markers and its size was previously estimated as 70 kb (San Millan et al., 1985).

Here we report the complete sequence of pMccB17, with some comparative genomic analysis. This sequence provides an insight into the biology of a prototypical microcin plasmid and a definitive sequence for the *mcb* microcin operon

## 2 MATERIALS AND METHODS

### 2.1 Bacterial strains and plasmids

The *E. coli* K‐12 strains ZK0005 (RYC1000 [pMccB17]) and ZK359 (MC4100 [pPY113]) were provided by Professor Roberto Kolter of Harvard Medical School.

### 2.2 DNA purification

Bacterial genomic DNA was isolated from strain ZK0005 as part of the initial DNA sequencing process as follows (this was carried out by microbesNG, details below). Cells were lysed in TE buffer containing lysozyme (final concentration 0.1 mg/mL) and RNase A (0.1 mg/mL) with incubation for 25 min at 37°C. Proteinase K (0.1 mg/mL) and sodium dodecyl sulfate (final concentration 0.5% v/v) were then added and this mixture was incubated for 5 min at 65°C. Genomic DNA was purified using an equal volume of solid‐phase reversible immobilization beads and resuspended in elution buffer (EB;10 mM Tris‐HCl, pH 8.5).

Plasmid DNA (pMccB17) was isolated from strain ZK0005 by alkaline lysis followed by anion‐exchange chromatography (Plasmid Midi kit, Qiagen) according to the manufacturer's recommendations for “very low‐copy” plasmids. DNA was eluted in EB buffer as above.

Plasmid DNA (pPY113) was isolated by alkaline lysis followed by chromatography using a silica matrix (Monarch Plasmid Miniprep Kit, New England Biolabs. DNA was eluted in DNA elution buffer (10 mM Tris‐HCl, 0.1 mM EDTA, pH 8.5).

### 2.3 DNA sequencing

Plasmid pMccB17 was sequenced twice using an Illumina short‐read next‐generation method by microbesNG (http://www.microbesng.com). First, the plasmid was sequenced together with the *E. coli* host bacterial chromosome; subsequently, plasmid DNA was purified as described above and sequenced separately. Genomic DNA libraries were prepared using the Nextera XT Library Prep Kit (Illumina) following the manufacturer's protocol with the following modifications: input DNA was increased twofold and polymerase chain reaction (PCR) elongation time was increased to 45 s. DNA quantification and library preparation were carried out on a Hamilton Microlab STAR automated liquid handling system (Hamilton Bonaduz AG). Libraries were sequenced on an Illumina NovaSeq. 6000 (Illumina) using a 250 bp paired‐end protocol. Reads were adapter trimmed using Trimmomatic version 0.30 (Bolger et al., 2014), with a sliding window quality cutoff of Q15. De novo assembly was performed on samples using SPAdes version 3.7 (Bankevich et al., 2012) and contigs were annotated using Prokka version 1.11 (Seemann, 2014).

Plasmid pPY113 was sequenced using a nanopore platform (PromethION with v14 chemistry and R10.4.1. flow cells; Oxford Nanopore Technologies). Sequencing and annotation were performed by Plasmidsaurus.

Sanger DNA sequencing of PCR amplicons, for plasmid genome finishing/gap resolution, was carried out by DBS Genomics (Durham University, UK).

### 2.4 Plasmid sequence gap closing

PCR Primers were designed to amplify across gaps in the draft sequence, and the corresponding oligonucleotides were synthesized by Integrated DNA Technologies (Appendix Table A1). PCR amplification used ImmoMix (Bioline) or Q5 High Fidelity (New England Biolabs) PCR master mixes, following the manufacturer's recommendations. Thermal cycling was carried out in a T100 thermal cycler (BioRad) and amplified products were electrophoresed in 1% agarose gels at 6 V/cm for 1 h, stained with GelRed, and visualized using a ChemiDoc XRS+ imaging system (BioRad). Products of the expected sizes were excised, and gel‐purified using Monarch DNA Gel Extraction Kit (NEB) and then sequenced (Sanger method, as above).

### 2.5 Annotation of pMccB17

The draft genome sequence was annotated automatically by MicrobesNG using Prokka 1.11 (Seemann, 2014). These annotations were manually checked using BLASTn (Zhang et al., 2000) and BLASTp (Altschul, 1997) retaining the default parameters. Coding sequences (CDSs) without proper annotation were manually assigned one where possible, using the BLAST result as a guide and Artemis to edit the annotation (Berriman, 2003; Rutherford et al., 2000).

### 2.6 pMccB17 plasmid replicon and conjugation system typing

Replicon typing was carried out using the IncF RST (replicon sequence typing) scheme, as implemented by pubMLST (https://pubmlst.org/plasmid/; Jolley et al., 2018) and pMLST (https://cge.cbs.dtu.dk/services/pMLST/; Carattoli et al., 2014). Initial classification of the relaxase was carried out using MOBscan (https://castillo.dicom.unican.es/mobscan/; Garcillán‐Barcia et al., 2020) and manually refined by pairwise comparisons with selected relaxase proteins using EMBOSS Needle (https://www.ebi.ac.uk/Tools/psa/emboss_needle/; Needleman & Wunsch, 1970).

### 2.7 Phylogenetic tree building

The phylogenetic tree of ParB‐fusion (Pbf) protein homologs was built using a maximum‐likelihood method as implemented by MEGA X (Kumar et al., 2018). The tree used the LG + G model (Le & Gascuel, 2008) for amino acid substitution.

### 2.8 Identification of resistance genes, insertion sequence, and virulence factors

Identification of resistance genes was performed by submitting the complete plasmid nucleotide sequence to the ResFinder web server (https://cge.cbs.dtu.dk//services/ResFinder/; Zankari et al., 2012). The nucleotide sequence was also submitted to the IS‐Finder web server (https://www.issaga.biotoul.fr/; Varani et al., 2011), and VFDB web server (https://www.mgc.ac.cn/VFs/; Liu et al., 2019) to identify insertion sequences and virulence factors, respectively.

## 3 RESULTS AND DISCUSSION

### 3.1 Plasmid pMccB17 genome assembly

Draft genomes were obtained using a short‐read, high coverage (Illumina) approach as described in Methods. Plasmid contigs from the draft genome were scaffolded as follows. Two contigs, 1.27 and 1.52, from the first draft sequence (of plasmid and chromosomal DNA), were discovered to have standard plasmid‐related features by manually examining the genome sequence. Contig 1.27 (60,265 bp) had 71 genes including the MccB17 operon, conjugative transfer system, replicon system, and some plasmid maintenance genes (*stbA, stbB, parE*), whereas contig 1.52 (8894 bp) had 12 genes, half of which have plasmid maintenance related functions, that is, *ssb, pbf, psiA, psiB*, and *hok/sok*. Contig 2.1, the first contig in the second draft sequence (from purified plasmid DNA), contained all the genes present in contigs 1.27 and 1.52, and when the three contigs were aligned using Artemis Comparison Tool (Carver et al., 2005, 2008), it was evident that contigs 1.27 and 1.52 make up contig 2.1. The ends of contig 2.1 were found to be within a CDS, gene_81, found in contig 1.27. PCR primers were designed to flank gaps in the plasmid sequence and were employed to close these gaps by PCR amplification followed by Sanger sequencing of the amplicons.

Finally, 114 nucleotides missing from contig 2.1 were added manually using Artemis, resulting in a complete circular genome sequence for pMccB17. This sequence was submitted to GenBank (accession number ON989342).

### 3.2 Plasmid replicon and conjugation system typing

The replicon sequence type was assigned using the IncF RST scheme as implemented by pubMLST and pMLST, indicating a FAB type of F2:A‐:B‐; so pMccB17 has an FII replicon (allele 2) without additional FIA or FIB replicons (Villa et al., 2010). Initial classification of the relaxase using MOBscan assigned it to the MOB_F_ family. The first 300 N‐terminal amino acids of the TraI relaxase/helicase protein (that is, the relaxase domain) were then compared in a pairwise manner with archetypal IncF plasmid relaxases. The pMccB17 relaxase domain was 99.3% identical to that of the IncFII plasmid R100 (GenBank: NC_002134) and 91.3% identical to that of F plasmid (GenBank: NC_002483), placing it in the MOB_F121_ type (Garcillán‐Barcia et al., 2011), or group A according to a recent phylogeny of IncF relaxases (Fernandez‐Lopez et al., 2016).

### 3.3 Plasmid pMccB17 genome analysis overview

**Figure 1:**
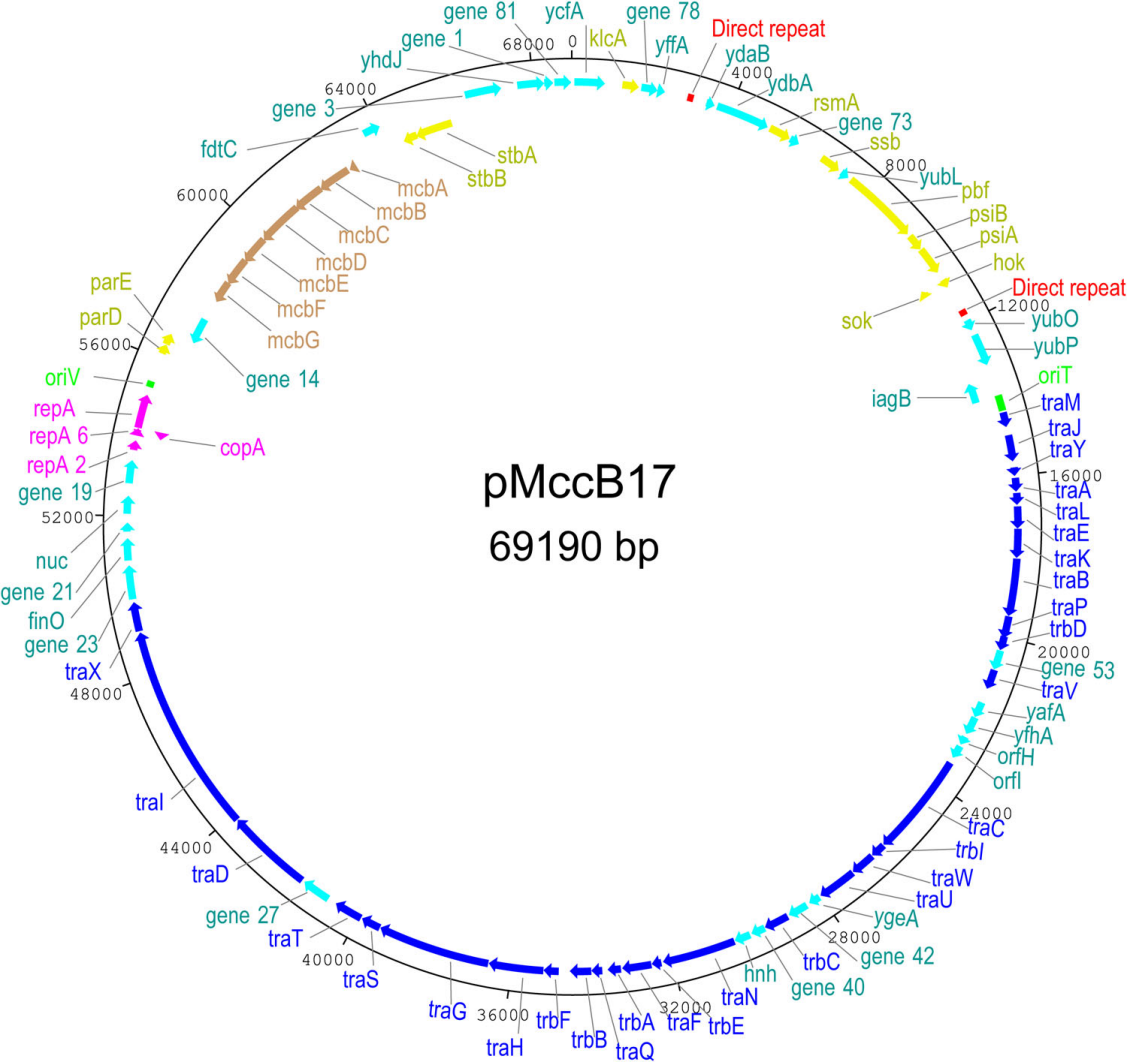
Circular Map of pMccB17. The outer ring shows the size of the plasmid, each tick representing 4 kb. The microcin operon is shown in brown, plasmid maintenance systems are shown in yellow, the replicon is shown in pink the conjugative transfer system is represented in blue, hypothetical proteins are shown in turquoise, origins of replication are in green and the 149 bp direct repeats are shown in red. Analysis based on the complete sequence as submitted to Genbank (ON989342). Diagram generated using DNAplotter (Carver et al., 2009).

pMccB17 is a circular IncFII plasmid (69,190 bp) with an average GC content of 51% and 83 CDS (Figure 1 and Appendix Table A2). This plasmid encodes MccB17, having the MccB17 operon. The *tra* region (*traMJYALEKBPVCWUNFQHGSTDIX* and *trbDICEABF*) encodes conjugative transfer functions and takes up about half of the plasmid backbone. pMccB17 encodes two types of toxin–antitoxin (TA)/plasmid addiction system, for eliminating plasmid‐free segregants. A type I *hok*/Sok system is located downstream of *psiA* and a type II system encoding ParDE is encoded downstream of the replication gene *repA*. The plasmid has no known antibiotic resistance genes, virulence factors, or transposable elements, according to ResFinder, IS‐Finder, and VFDB web servers, respectively.

### 3.4 MccB17 operon

The *mcb* operon, consisting of genes *mcbABCDEFG* encoding biosynthetic and immunity functions for MccB17, is located between *gene_14* (encoding a hypothetical protein) and *fdtC* (encoding an acetyltransferase). The nucleotide and amino acids sequences of genes in the *mcb* operon of pMccB17 (ON989342) were compared with some historical published sequences (Table 1). The M24253 (Genbank) sequence, derived by Sanger sequencing of a cloned DNA fragment of pMccB17, provided the first available sequence of *mcbA, mcbB, mcbC*, and *mcbD* (Genilloud et al., 1989). The X07875 (Genbank) sequence, also obtained via Sanger sequencing of a cloned fragment of pMccB17, includes the remaining three genes *mcbE, mcbF*, and *mcbG* (Garrido et al., 1988). The ON989342 sequence should in theory be identical to the M24253 and X07875 sequences and the plasmid pMccB17 as sequenced here was provided by one of the publishing authors (Roberto Kolter) of the historical sequences (Garrido et al., 1988; Genilloud et al., 1989). The FM877811 sequence provides a complete *mcb* operon sequence and was selected because of this. This is not the original *mcb* operon, rather it is from the whole genome sequence of *E. coli* strain L1000, isolated from human feces, and appears to be chromosomal rather than plasmid‐borne (Zihler et al., 2009). Nucleotide sequences of *mcbA, mcbC, mcbE*, and *mcbG* were 100% identical to those in the historical sequences M24253 and X07875. However, *mcbB* and *mcbD* each differed by a single base pair from M24253, resulting in a single nonconservative amino acid substitution in each case (the amino acid in our sequence is shown first): S117C for McbB and R171T for McbD. Our McbB and McbD sequences also differed from FM877811 by a single (but different) substitution in each case: E198D and A113T respectively. As sequenced here, *mcbF* was annotated as 732 bp versus 744 bp in the historical X07875 sequence, reflecting a frameshift towards the 3′ end of the latter gene due to the C at position 688 of the new sequence is missing from X07875. There are also five substitutions in the sequence 5′ of this indel and overall this gene and hence the encoded McbF protein has the greatest difference from historical published sequences for the *mcb* operon (Table 1).

**Table 1:** Comparison of the proteins encoded in the *mcb* operon of pMccB17 (ON989342) with selected published *mcb* operon sequences.

Protein	Predicted function	Number of amino acids (% identity to ON989342)			
		GB:ON989342	GB:FM877811	GB:M24253	GB:X07875
McbA	Microcin precursor	69	69 (100)	69 (100)	−
McbB	Microcin B synthase enzyme complex	295	295 (99.7)	295 (99.7)	−
McbC		272	272 (100)	272 (100)	−
McbD		396	396 (99.7)	396 (99.7)	−
McbE	Self‐immunity and export	241	241 (100)	−	241 (100)
McbF	Self‐immunity and export	243	243 (100)	−	247 (91.5)
McbG	Self‐immunity	187	187 (99.5)	−	187 (100)

We do not believe that the RYC1000 [pMccB17]) strain sequenced here has been passaged extensively since it was derived from the original capture of pMccB17 by conjugation into BM21 (Baquero et al., 1978). Due to the time elapsed, we are unable to confirm how many passages the pMccB17 plasmid has undergone between the original sequencing and our sequencing reported here. It is possible that the differences we observed, compared to the original sequences, are due to mutations accumulated during passage. However, we hypothesized that the differences observed were due to Sanger sequencing errors in the historical sequences i.e. that our pMccB17 sequence is correct. To confirm the *mcb* operon sequence presented here, we obtained pPY113 another clone of the *mcb* operon that was independently derived from the parental BM21 [pMccB17] strain, and sequenced it using an orthogonal high‐coverage long‐read approach (Yorgey et al., 1994). The resulting pPY113 plasmid sequence (Genbank: OR091272) is identical to our sequence of pMccB17 throughout the shared *mcb* operon sequences. Hence, we believe that it is unlikely that these differences reflect mutations accumulated by our pMccB17 strain and much more likely that they are due to inaccuracies in Sanger sequencing, which is a relatively error‐prone process.

### 3.5 Direct repeats

**Figure A1:**
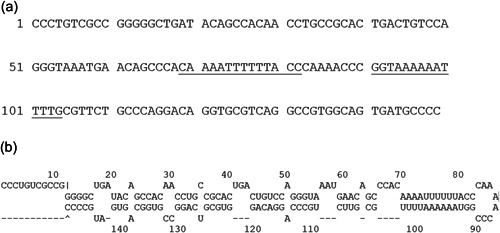
Direct repeat sequence from pMccB17. This sequence occurs twice in the pMccB17 genome. (a) The 149 bp repeated sequence. The two arms of a large perfect palindrome at the center of the sequence have been underlined. (b) Predicted secondary structure formed by RNA encoded by this sequence (initial Δ*G* = −67.80). A highly similar structure was predicted for ssDNA (dG = −35.09). These structures were predicted using mfold version 3.6, with default parameters, as implemented by the UNAFold Web Server (Zuker, 2003; www.unafold.org).

In the process of aligning contigs 1.27 and 1.52 with contig 2.1, to ascertain the complete plasmid sequence, we discovered a 149 bp direct repeat in contig 2.1 (Appendix Figure A1). This 149 bp sequence is repeated twice (8639 bp apart) in the sequence of pMccB17. These repeats are identical and each has an intragenic location: one is located between *yffA* and *ydaB*, whereas the other is located between *hok*/*sok* and *yubO*. These repeats have 12 genes in between them, including *ssb, pbf, psiAB*, and the *hok/sok* TA system. This approximately corresponds to the plasmid leading region as originally defined for F plasmid (Loh et al., 1989). A large perfect palindrome [5′‐CAAAATTTTTTACC]CAAAACCC[GGTAAAAAATTTTG‐3′] is present at the center of this sequence, with some imperfectly palindromic sequences to either side (Appendix Figure A1). This may result in the formation of functional secondary structures, in either single‐stranded DNA produced during conjugation or mRNA produced during transcription.

Searches using BLASTn against the nucleotide collection (nr/nt) database at NCBI revealed that the repeated sequence is present in IncF plasmids of Gammaproteobacteria, mainly from the Enterobacterales, In 20 plasmids examined, having query coverage and identity of 100%, copy number ranged from 1 to 3 and all were members of the IncF family. The exact function of these repeated sequences is unknown, but several genes are commonly flanked by these repeats, including *ssb, pbf* (see below), and *psiAB*.

### 3.6 Pbf protein

**Figure A2:**
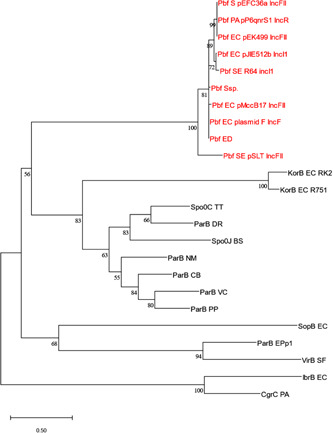
Phylogenetic analysis of ParB family proteins. Pbf proteins are highlighted in red. The clades are named in this order: name of the ParB family protein, two letters representing the organism it is found, plasmid it is found in (only in Pbf), and plasmid incompatibility group (only in Pbf). EC = *Escherichia coli*; SE = *Salmonella enterica*; S = *Salmonella*; PA = *Pseudomonas aeruginosa*; Ssp. = *Streptomyces* sp.; ED = *Enterococcus durans*; TT = *Thermus thermophilus*; BS = *Bacillus subtilis*; EPp1 = *Escherichia phage* P1; SF = *Shigella flexneri*; CB = *Coxiella burnetii*; DR = *Deinococcus radiodurans*; VC = *Vibrio cholerae*; NM = *Neisseria meningitidis*; PP = *Pseudomonas putida*. The evolutionary history was inferred by using the Maximum Likelihood method and the Le_Gascuel (L + G) model. The tree with the highest log likelihood (−5208.41) is shown. The percentage of trees in which the associated taxa clustered together is shown below the branches. Initial tree(s) for the heuristic search were obtained automatically by applying Neighbor‐Join and BioNJ algorithms to a matrix of pairwise distances estimated using a JTT model and then selecting the topology with superior log likelihood value. A discrete Gamma distribution was used to model evolutionary rate differences among sites (5 categories ( + *G*, parameter = 3.2901)). The tree is drawn to scale, with branch lengths measured in the number of substitutions per site. All positions with less than 95% site coverage were eliminated, that is, fewer than 5% alignment gaps, missing data, and ambiguous bases were allowed at any position (partial deletion option). There were a total of 169 positions in the final data set. Evolutionary analyses were conducted in MEGA X.

Upstream of *psiB* is a gene encoding 652 amino acids that we have annotated as “*pbf*” (for **P**ar**B f**usion) as it features a ParB‐like N‐terminal domain joined to a C‐terminal region that does not include any known conserved domain. A BLASTp search of its amino acids against the nonredundant protein sequences (nr) database at NCBI confirmed that its N‐terminal region (the first 250 amino acids) contains a conserved domain, annotated as “ParB/RepB/Spo0J family partition protein.” Thus, Pbf is evolutionarily, if not functionally, related to proteins that (via interactions with an NTPase partner ParA and a centromere‐like partition site on the DNA, *parS*) are involved in the active partitioning of bacterial chromosomes and low copy‐number plasmids (McLean & Le, 2023; Appendix Figure A2). The classical plasmid F carries a homologous gene (*orf652* which is 94% identical to *pbf*) and the similarity of the encoded protein to ParB was first described by Manwaring et al. (1999).

## 4 CONCLUSIONS

Plasmid pMccB17 seems a typical member of the IncFII family, apart from its carriage of the MccB17 biosynthetic gene cluster *mcb*. This plasmid does not carry any identifiable insertion sequence or other mobile genetic elements, nor does it encode any known antibiotic resistance genes (apart from those conferring immunity to MccB17) or pathogenicity factors. We have reported here a complete and accurate sequence of the *mcb* operon, which will be useful for future studies and manipulation of the biosynthetic pathway for this prototypical RiPP.

## AUTHOR CONTRIBUTIONS

**Mayokun Ajeigbe**: Conceptualization (equal), writing—original draft (lead), formal analysis (lead), writing—review and editing (equal). **Stephen Childs**: Conceptualization (equal), writing—review and editing (equal). **Timothy Paget**: conceptualization (equal), writing—review and editing (equal). **Lewis Bingle**: Conceptualization (lead), formal analysis (supporting), writing—review and editing (equal).

## CONFLICT OF INTEREST STATEMENT

The authors declare no conflict of interest.

## ETHICS STATEMENT

None required.

## Data Availability

Plasmid genome sequences are available in GenBank with the following accession numbers: pMccB17, ON989342: https://www.ncbi.nlm.nih.gov/nuccore/ON989342; and pPY113, OR091272: https://www.ncbi.nlm.nih.gov/nuccore/OR091272

## 1

**Table A1:** Primer sequences for gap‐closing PCR amplifications between contigs of pMccB17.

Gaps	5′ Forward 3′	5′ Reverse 3′	Annealing temperature (°C) and PCR system
Contig 1.27	CTGATACAGCCACAACCTGC	GTTTGAGAACCTTCGCCGC	65, Q5 Master Mix
Contig 1.52	CTGATACAGCCACAACCTGC	CTGTTTGAGAACCTTCGCCG	65, Q5 Master Mix
Contig 2.1	AGAAATACCGGATTCAGTCCG	GAAATCAGGACTGTCCTGGC	50‐60, ImmoMix

Abbreviation: PCR, polymerase chain reaction.

**Table A2:** CDS identified in pMccB17.

CDS	Position (bp)	Protein function
*ycfA*	49. .819	Hypothetical protein
*klcA*	1235. .1660	Antirestriction protein
*gene_78*	1707. .2129	Hypothetical protein
*yffA*	2126. .2317	Hypothetical protein
*ydaB*	3355. .3585	Hypothetical protein
*ydbA*	3637. .4998	Hypothetical protein
*rsmA*	5045. .5608	Ribosomal RNA small subunit methyltransferase A
*gene_73*	5608. .5874	Hypothetical protein
*ssb*	6517. .7056	Plasmid‐derived single‐stranded DNA‐binding protein
*yubL*	7118. .7351	Hypothetical protein
*pbf*	7416. .9374	ParB fusion protein
*psiB*	9429. .9863	Plasmid SOS inhibition protein B
*psiA*	9860. .10579	Plasmid SOS inhibition protein A
*sok*	Complement (10748. .10813)	Antisense RNA of hok transcripts
*mok*	10801. .11013	Modulation of hok protein
*hok*	10859. .11017	Postsegregation killing protein
*yubO*	11944. .12231	Hypothetical protein
*yubP*	12350. .13171	Hypothetical protein
*iagB*	Complement (13466. .13975)	Invasion protein
*traM*	14391. .14774	Relaxosome protein
*traJ*	14968. .15639	Conjugal transfer transcriptional regulator
*traY*	15776. .16003	Relaxosome protein TraY
*traA*	16036. .16395	Pilin TraA
*traL*	16410. .16721	Peripheral membrane protein TraL
*traE*	16743. .17309	Bacterial sex pilus assembly and synthesis proteins TraE
*traK*	17296. .18024	Pilus assembly TraK
*traB*	18024. .19475	Involved in F pilus assembly
*traP*	19465. .20031	Conjugation protein
*trbD*	19970. .20341	Conjugal transfer protein TrbD
*gene_53*	20334. .20843	Hypothetical protein
*traV*	20836. .21348	Conjugative transfer system lipoprotein
*yafA*	21697. .22110	Hypothetical protein
*yfhA*	22103. .22576	Hypothetical protein
*orfH*	22657. .22875	Hypothetical protein
*orfI*	22903. .23250	Hypothetical protein
*traC*	23376. .26006	Conjugative transfer ATPase
*trbI*	26003. .26389	Type‐F conjugative transfer system protein
*traW*	26386. .27018	Type‐F conjugative transfer system protein
*traU*	27015. .28007	Conjugal DNA transfer protein
*ygeA*	28034. .28342	Hypothetical protein
*gene_42*	28405. .28923	Hypothetical protein
*trbC*	28950. .29588	Type‐F conjugative transfer system pilin assembly protein
*gene_40*	29585. .29956	Hypothetical protein
*hnh*	29981. .30403	Hnh nuclease
*traN*	30400. .32250	Type‐F conjugative transfer system mating‐pair stabilization protein
*trbE*	32277. .32534	Type IV conjugative transfer system protein
*traF*	32527. .33270	Type‐F conjugative transfer system pilin assembly protein
*trbA*	33284. .33628	Conjugal transfer repressor
*traQ*	33747. .34031	Type‐F conjugative transfer system pilin chaperone
*trbB*	34018. .34563	Type‐F conjugative transfer system pilin assembly thiol‐disulfide isomerase
*trbF*	34821. .35213	Plasmid mobilization
*traH*	35200. .36573	Type IV conjugative transfer system protein
*traG*	36570. .39395	Mating pair stabilization protein
*traS*	39392. .39901	Conjugal transfer entry exclusion protein
*traT*	39915. .40646	Lipoprotein TraT
*gene_27*	40849. .41586	Hypothetical protein
*traD*	41637. .43835	Type IV conjugative transfer system, coupling protein TraD
*traI*	43835. .49105	Conjugative transfer relaxase protein
*traX*	49125. .49871	Type‐F conjugative transfer system pilin acetylase
*gene_23*	49930. .50790	2,6‐dihydropseudooxynicotine hydrolase
*finO*	50893. .51453	Fertility inhibition protein
*gene_21*	51598. .51840	Hypothetical protein
*nuc*	52039. .52500	Thermonuclease nuc
*gene_19*	52794. .53384	Hypothetical protein
*repA2*	53624. .53881	Replication regulatory protein
*copA*	Complement (54012. .54104)	Leader peptide regulator for RepA expression
*repA6*	54116. .54190	
*repA*	54183. .55040	Plasmid replication initiator
*relB/parD*	56110. .56373	Toxin‐antitoxin system
*relE/parE*	56363. .56662	Toxin‐antitoxin system
*gene_14*	Complement (56698. .57363)	Hypothetical protein
*mcbG*	Complement (57933. .58496)	Microcin B17 immunity protein, McbG
*mcbF*	Complement (58500. .59231)	Microcin B17 export protein, McbF
*mcbE*	Complement (59228. .59953)	Microcin B17 export protein, McbE
*mcbD*	Complement (59963. .61153)	Microcin B17 synthetase complex protein, McbD
*mcbC*	Complement (61134. .61952)	Microcin B17 synthetase complex protein, McbC
*mcbB*	Complement (61954. .62841)	Microcin B17 synthetase complex protein, McbB
*mcbA*	Complement (62868. .63077)	Microcin B17 precursor peptide
*fdtC*	63607. .64059	dTDP‐3‐amino‐3,6‐dideoxy‐alpha‐d‐galactopyranose 3‐N‐acetyltransferase
*stbB*	Complement (64462. .64815)	Type IV partitioning system
*stbA*	Complement (64815. .65777)	Type IV partitioning system
*gene_3*	66289. .67215	Hypothetical protein
*yhdJ*	67600. .68283	DNA adenine methyltransferase YhdJ
*gene_1*	68284. .68505	Hypothetical protein
*Gene_81*	68519. .68950	Hypothetical protein

Abbreviation: CDS, coding sequence.
